# Leukoaraiosis Significantly Worsens Driving Performance of Ordinary Older Drivers

**DOI:** 10.1371/journal.pone.0108333

**Published:** 2014-10-08

**Authors:** Kimihiko Nakano, Kaechang Park, Rencheng Zheng, Fang Fang, Masanori Ohori, Hiroki Nakamura, Yasuhiho Kumagai, Hiroshi Okada, Kazuhiko Teramura, Satoshi Nakayama, Akinori Irimajiri, Hiroshi Taoka, Satoshi Okada

**Affiliations:** 1 Interfaculty Initiative in Information Studies, The University of Tokyo, Tokyo, Japan; 2 Traffic Medicine Laboratory, Research Organization for Regional Alliances, Kochi University of Technology, Kami-shi, Kochi, Japan; 3 The Brain Checkup Center, Kochi Kenshin Clinic, Kochi-shi, Kochi, Japan; 4 Institute of Industrial Science, The University of Tokyo, Tokyo, Japan; 5 Faculty of Engineering, Kanagawa University, Yokohama, Japan; 6 Regional ITS Research Laboratory, Research Organization for Regional Alliances, Kochi University of Technology, Kami-shi, Kochi, Japan; 7 Driver's License Center, Kochi Prefectural Police Department, Ino-cho, Kochi, Japan; University of Akron, United States of America

## Abstract

**Background:**

Leukoaraiosis is defined as extracellular space caused mainly by atherosclerotic or demyelinated changes in the brain tissue and is commonly found in the brains of healthy older people. A significant association between leukoaraiosis and traffic crashes was reported in our previous study; however, the reason for this is still unclear.

**Method:**

This paper presents a comprehensive evaluation of driving performance in ordinary older drivers with leukoaraiosis. First, the degree of leukoaraiosis was examined in 33 participants, who underwent an actual-vehicle driving examination on a standard driving course, and a driver skill rating was also collected while the driver carried out a paced auditory serial addition test, which is a calculating task given verbally. At the same time, a steering entropy method was used to estimate steering operation performance.

**Results:**

The experimental results indicated that a normal older driver with leukoaraiosis was readily affected by external disturbances and made more operation errors and steered less smoothly than one without leukoaraiosis during driving; at the same time, their steering skill significantly deteriorated.

**Conclusions:**

Leukoaraiosis worsens the driving performance of older drivers because of their increased vulnerability to distraction.

## Introduction

The issue of older drivers has become an important topic in the modern traffic environment, and researchers have paid great attention to traffic crashes related to older drivers [1]–[3]. The effects of brain disease on driver performance have also been investigated in recent years [4]–[6]. In part, this focus indicates that there is a possible relationship between driver performance and the quality of brain tissue, especially for older drivers. However, up to now, there has been little research to explain driver performance in terms of changes in the brain tissue of ordinary drivers.

Leukoaraiosis (LA) describes vacuous lesions full of water produced by damaged brain tissue in the white matter of the brain that acts as connectors through neuronal fibers [7]. LA has been reported to increase in frequency according to aging and to be significantly associated with hypertension, diabetes, hyperlipidemia, and metabolic syndrome [8]. Based on examinations using magnetic resonance imaging (MRI) for atherosclerosis risk in communities, LA was classified into three grades, which range from G0 to G2 [9]. Typically, G0 is without subcortical LA, G1 is with tiny subcortical LA, mainly concerning the unilateral cerebral hemisphere, and G2 is with multiple subcortical LA mainly concerning the bilateral hemispheres. A significant association between LA found using the MRI method and traffic crashes has been presented in our previous study [10]. However, the effects of LA on driver performance still remain unclear, and more work needs to be done to address this issue.

In this experimental study, differences in brain tissue for each participant were examined using MRI, and classified based on the existence of leukoaraiosis progression over the white matter in bilateral cerebral hemispheres. The driving performance of participants with LA or without LA was evaluated using driving operation errors and the amount of rough steering under the distracted condition of both driving and calculating.

## Experiments

### Participants

**Figure 1:**
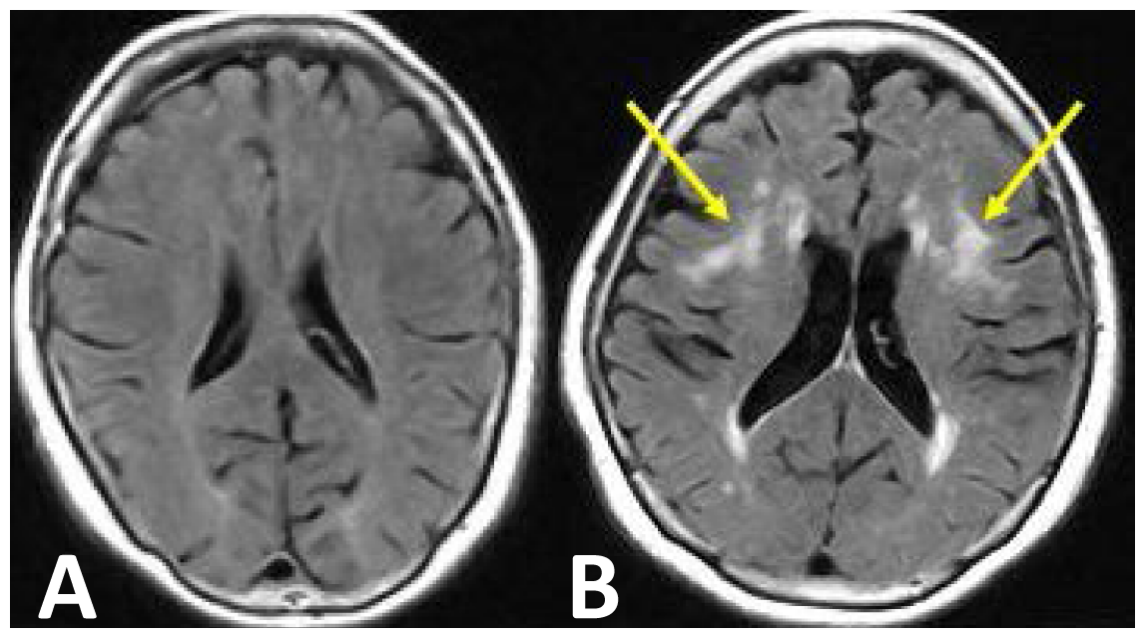
Typical MRI of G0 (right panel) and G2 (left panel; arrows indicate leukoaraiosis).

Thirty-three participants took part in the driving experiment, none of whom had had symptomatic cerebral diseases such as cerebral stroke. They were split into three groups: Young (20 s), N = 9, aged 24.7±3.6 years (mean ± S.D.), driving frequency of 12.4±12.7 times per month, with a degree of LA of G0 at the time of the driving experiment; Older G0 (G0), N = 11, aged 69.9±6.6 years, driving frequency of 19.6±10.4 times per month, with a degree of LA of G0 at the time of the driving experiment; and Older G2 (G2), N = 13, aged 69.5±6.1 years, driving frequency of 20.8±10.4 times per month, with a degree of LA of G2 at the time of the driving experiment. Typical MRIs of the G0 and G2 groups are shown in Figure 1.

The classification of LA for all participants was checked using MRI, by two neurosurgeons following the normal procedure of the Kochi Health Checkups Center of Japan, and an ethics examination was carried out before the MRI check. The driving experiment was performed with the consent of the 33 participants, and a formal agreement to participate in the experiments was signed by all participants, outlining that the experimental data would only be used for scientific study, and that the results would ensure anonymity. This study was approved by the institutional review board at Kochi University of Technology.

### Driving Course

**Figure 2:**
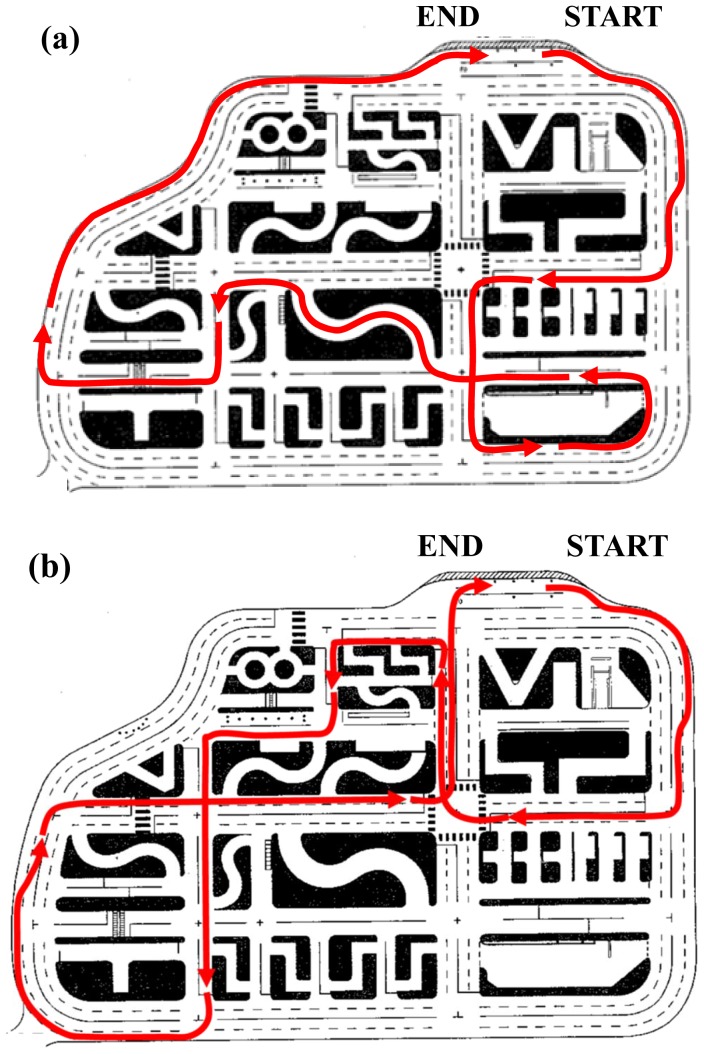
Diagram of the designated route at the testing center.

Actual-vehicle driving experiments were performed on a standard testing course in the Driver's License Center of Kochi Prefectural Police Department, Japan. Two driving routes were designed in this driving experiment. The driving route A included a slalom route in the center part of the Driver's License Center, and the driving route B did not include the slalom route (see Figures 2(a) and 2(b)). The driving route A was used for the ten participants, including two 20 s, four G0, and four G2. The driving course B was used for the other 22 participants. All of the participants used the identical experimental contents and processes in their driving experiment.

As shown in the figures 2(a) and 2(b); a driving route was designated from the start point, and drivers had to return to the start point following the red arrows. The driving route included a right turn lane, temporary stop signs, right-of-way, an intersection, and other standard traffic schemes. A traffic signal was installed in the intersections of two lanes going each way, and the signal was preliminarily set at a regulated time interval before the driving experiment. The drivers were instructed that the left lane should be chosen while driving on the two-lane road, and the speed restraint for this was 30 km/h. During the driving experiment, traffic pylons were used to mark the designated routes.

### Paced Auditory Serial Addition Test

**Figure 3:**
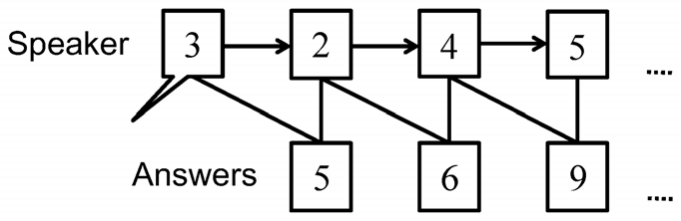
Flow of the paced auditory serial addition test.

The paced auditory serial addition test (PASAT) is often used as a sound load to investigate the effect of speech stimuli on driving performance [11]–[13]. In this study, the PASAT was used to induce the distraction of drivers' attention as an additional disturbance during the driving experiment [14]–[16]. As shown in Figure 3, a series of numbers, randomly generated from 0 to 5, were continuously announced by a speaker every 3 seconds during driving, and the participant was asked to immediately respond with the result of the currently heard number added to the previously heard number. The driver's answers were collected using a sound recorder (ICD-SX813 Sony, Japan), and the percentages of correct answers were calculated for every driving experiment.

In the beginning, each participant was allowed driving practice on the designated testing course, which included actual-vehicle driving with and without the PASAT. For the driving experiment, participants performed four trials of actual-vehicle driving with and without the PASAT. Considering the counterbalance problem and potential influence by the continuous PASAT tasks, the same sequence of four trials of actual driving with, without, with, and without the PASAT was used for every participant. The duration of each driving experiment was 10–15 minutes, and the resting time between the driving experiments was 5 minutes.

### Rating of Driver Skill

The driving skill of participants was evaluated using a driving operation test, supervised by an official driving license examiner from Kochi Prefectural Police Department, Japan. The rating of driving performance was identical to that used in driving license testing in Japan, and was rated as 1, 2, and 4 evaluation credits for operation errors, from high to low evaluation levels. The credits were related to how participants drove normally and avoided errors, mainly regarding the driving behaviors of departure, speed maintenance, security verification, braking actions, pathway change, travel in a straight line, and turning right or left, and the relative sense of stability inside the car.

Critical operation errors, which were traffic regulation offenses such as a stop sign violation, were recorded separately, and places where operation errors occurred were also recorded. The rating basis was the same for all participants.

### Measuring of Vehicle State

**Figure 4:**
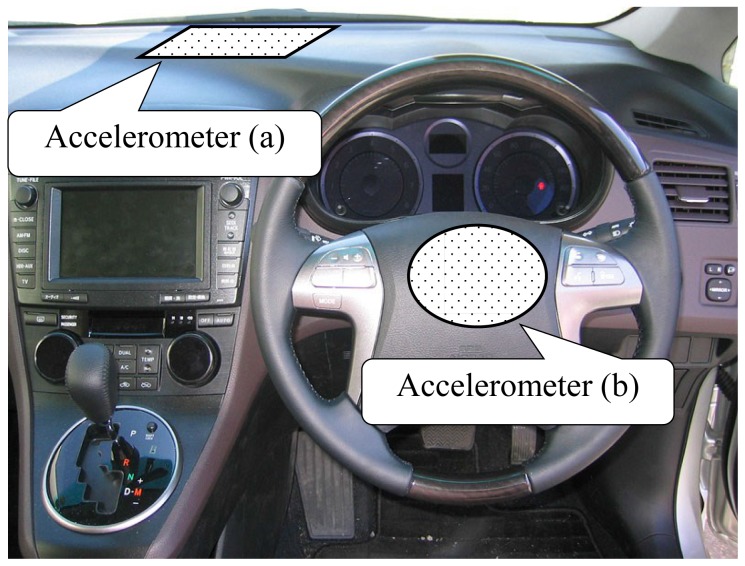
Positions of the 6-axis accelerometers in the car.

One motivation in this field of study is to investigate the characteristics of driving, through analysis of the steering capabilities of drivers with different degrees of LA. Therefore, two 6-axis accelerometers (Objet, ATR-Sensetech, Japan) were fixed to the inner body surface of the inner car (accelerometer (a)) and on the center of the steering wheel (accelerometer (b)). The 6-axis accelerating signals include the 3-axis accelerations on the X, Y, and Z axes, and three directional angular accelerations rotating around the X, Y, and Z axes. The fixed positions are shown in Figure 4. Accelerometer (a) was used to measure the rotation of vehicle relative to the ground, and accelerometer (b) was used to measure the rotation angle of steering.

### Steering Entropy Analysis

To analyze the steering operation based on the actual-vehicle driving experiment, we hypothesized that drivers would find it more difficult to drive smoothly when they were subjected to a sound load from the PASAT, resulting in distraction of attention. Referring to previous studies, steering entropy can be used to evaluate driving performance, driver workload and safety [17]–[21]; therefore, steering entropy was used in this study to estimate steering performance.

By the steering entropy method, the smoothness of drivers' steering was digitalized as an entropy value calculated from the time series of steering angle. For example, when the time interval of the measured data was 50 ms, the value of steering angle per 150 ms could be calculated from the mean value of three data points as a minimum control interval for the participant. Here, the steering angles for the last three data points were used to calculate the predicted steering angle *θ*_p_ (n) on the n^th^ time point using a quadratic Taylor expansion in the center of (n−1). The predicted error *e*(n) is the difference between *θ*_p_(n) and the steering angle *θ*(n) in real time at the n^th^ time point.

The frequency distribution *e*(n) can be derived from the driving data of the participant without the sound loading, and then the percentile value α at 90% can be calculated. If smooth steering is taking place, the central sharpness in the derived frequency distribution increases to an extent, and the *α* value also becomes small. The *α* value is an indicator of the participant's personal driving character, and is used as a criterion value when the driving load is added into the experimental conditions for the same participant. Based on the criterion value, the frequency distribution can be divided into nine cells, and the proportion for each cell can be estimated as P_1_, P_2_, …, P_9_. The entropy value (SEV) can be calculated by
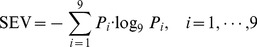
(1)


When the participant drives in the same driving conditions without loading, the frequency distribution can be estimated. Based on the cells of the estimated frequency distribution, the SEV in the loading condition can be calculated. Normally, the smoothness of steering operation becomes worse when the participant drives in the loaded condition, and the sharpness of the frequency also becomes worse with the increase of the SEV as a feature.

## Results

### Correct Answer Rates of the PASAT

**Figure 5:**
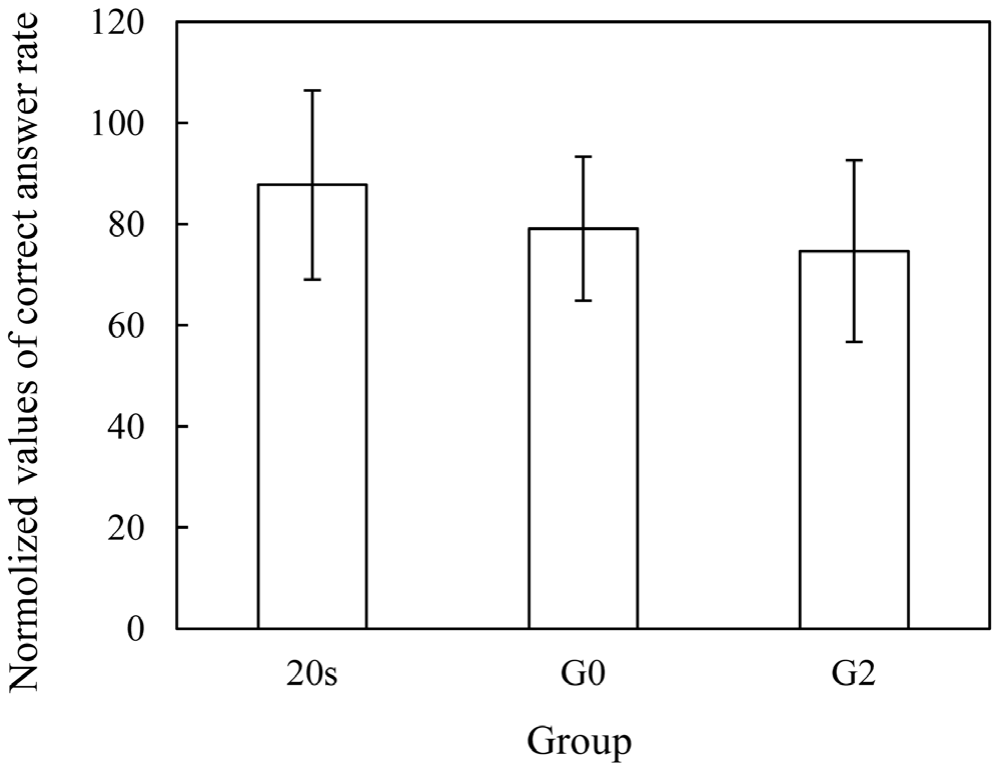
Normalized values of correct answer rates of PASAT (mean ± S.D.).

The correct answer rate of the PASAT, during the actual-vehicle driving experiment, was normalized by the correct answer rate in the preliminary testing, and result is shown in Figure 5. The values of correct answer rates decreased in turn from 20 s to G0 to G2, when the PASAT was used to induce the distraction of driver attention during the driving experiment. However, significant differences between the population means of the three groups were not found at the 0.05 level using a one-way ANOVA analysis.

### Rating of Driver Performance

**Figure 6:**
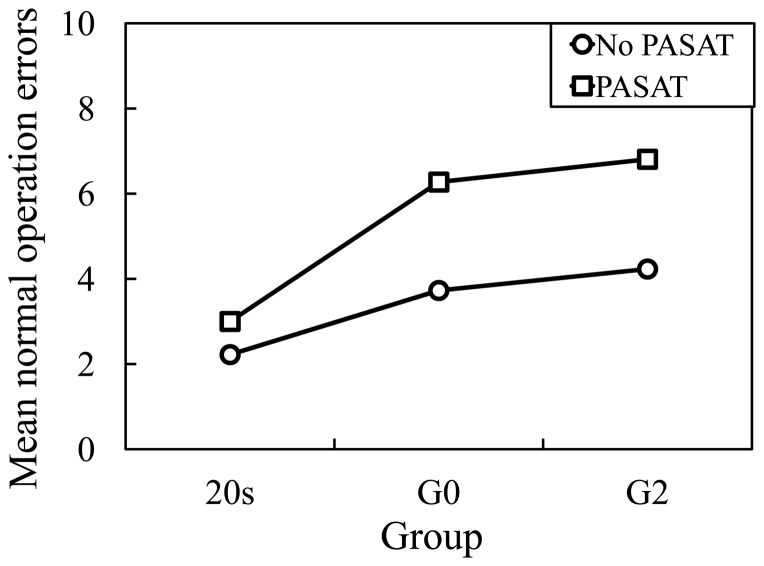
Normal operation errors.

**Figure 7:**
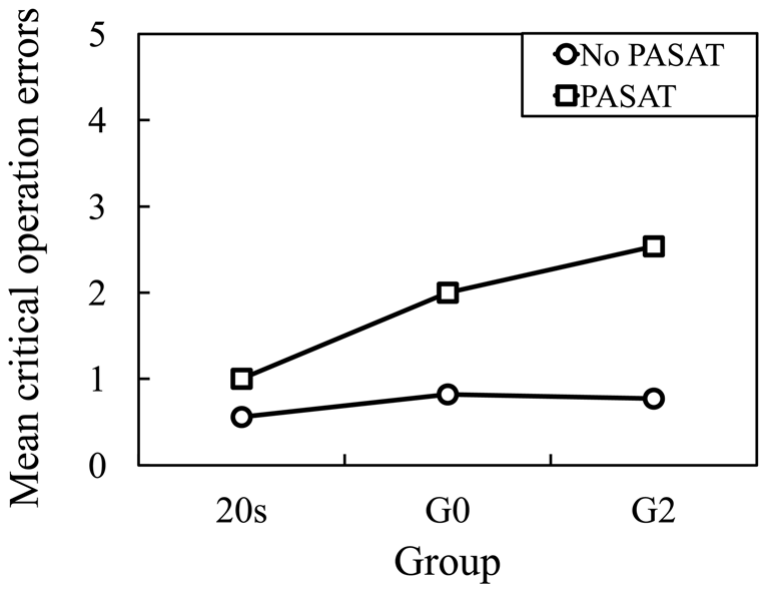
Critical operation errors.

The related results of the rating of driving performance are presented in Figures 6–7. A mixed factorial analysis of variance (ANOVA) that has a mixture of between-group and repeated measures variables was adopted for statistical analysis. In a two-way ANOVA, the between-group variables were related with the Age design (20 s×Go×G2) as the between-subjects factor, and the repeated measures variables are related with the PASAT design (PASAT×No PASAT) as the within-subjects factor.

For normal operation errors with and without PASAT (see Figure 6), there were a non-significant main effect of the Age, *F*(2, 30) = 1.97, *p* = 0.16, and a significant main effect of the PASAT, *F*(1, 30) = 30.64, *p*<0.001. Furthermore, there was a non-significant Age×PASAT interaction, *F*(2, 30) = 2.56, *p* = 0.09. By pairwise comparison, values of PASAT were significantly higher than that of No PASAT (*p*<0.001). Additionally, Turkey corrected post hoc tests showed that all values of 20 s and G0, 20 s and G2, and G0 and G2 did not significantly differ (*p* = 0.30, *p* = 0.15, and *p* = 0.93).

For critical operation errors with and without PASAT (see Figure 7), there were a significant main effect of the Age, *F*(2, 30) = 7.29, *p*<0.05, and a significant main effect of the PASAT, *F*(1, 30) = 61.50, *p*<0.001. Furthermore, there was a significant Age×PASAT interaction, *F*(2, 30) = 14.57, *p*<0.001. By pairwise comparison, values of PASAT were significantly higher than that of No PASAT (*p*<0.001). Additionally, Turkey corrected post hoc tests showed that values of 20 s and G0 did not significantly differ (*p* = 0.80), but values of G2 were significantly higher than both of 20 s and G0 (*p*<0.01 and *p*<0.05).

**Figure 8:**
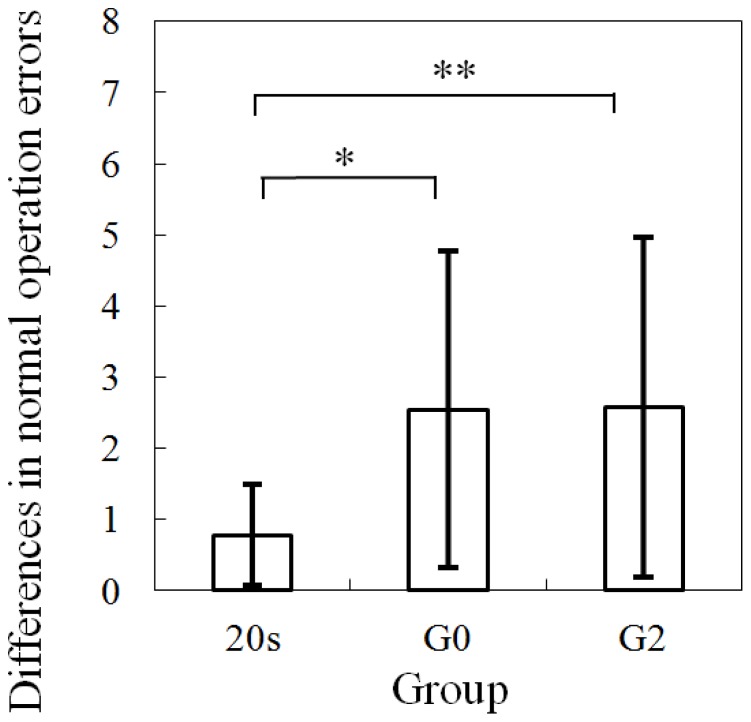
Differences in normal operation errors with and without PASAT (one-way ANOVA analysis: *F*(1, 18) = 5.18 and *p*<0.05; and *F*(1, 20) = 4.750 and *p*<0.05).

**Figure 9:**
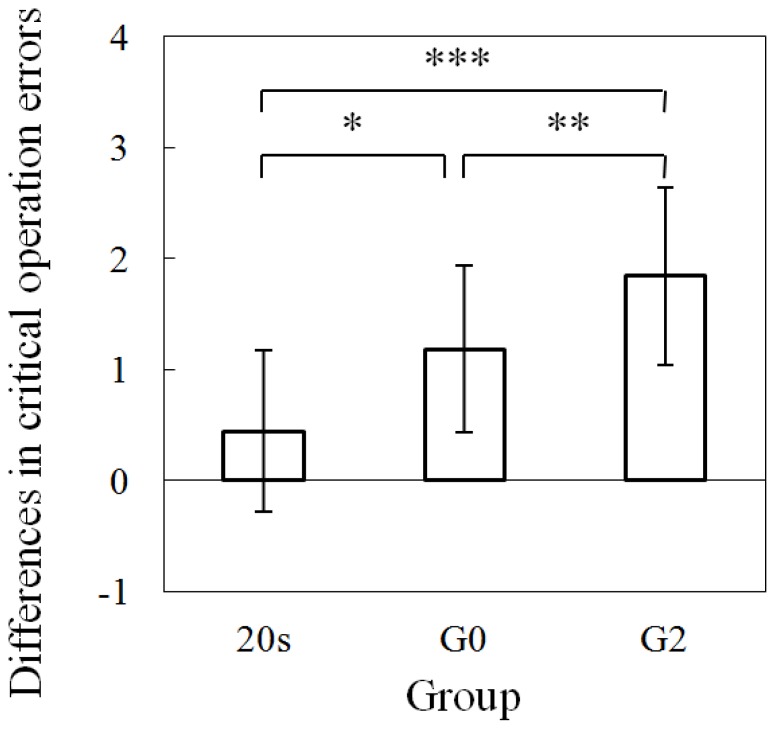
Differences in critical operation errors only for temporary stop with and without PASAT (one-way ANOVA analysis: * *F*(1, 18) = 4.91 and *p*<0.05; ** *F*(1, 22) = 4.34 and *p*<0.05; and *** *F*(1, 20) = 17.54 and *p*<0.001).

To further analyze the effects of PASAT loading on driving performance, the differences between the ratings of driver performance in the conditions with and without PASAT are presented in Figures 8 and 9. Normal operation error, as shown in Figure 8, showed significant differences at the 0.05 level between 20 s and G0, and between 20 s and G2 (one-way ANOVA analysis: *F*(1, 18) = 5.18 and *p*<0.05; and *F*(1, 20) = 4.75 and *p*<0.05). Non-temporary stop critical operation error, as shown in Figure 9, showed a significant difference at the 0.05 level between 20 s and G0 (one-way ANOVA analysis: *F*(1, 18) = 4.91 and *p*<0.05), and between 20 s and G2 (one-way ANOVA analysis: *F*(1, 20) = 17.54 and *p*<0.001). The above results indicate that the aging-related effect can cause the impairment of driving performance for both normal and critical operation errors.

Furthermore, there was a significant difference at the 0.05 level between G0 and G2 (one-way ANOVA analysis: *F*(1, 22) = 4.34 and *p*<0.05). Therefore, LA significantly worsens the driving performance of ordinary older drivers, which is dependent on the operating characteristics of the older drivers for temporary stops, even for the same age distributions as the G0 and G2 older drivers (see Participants).

### Results of Steering Entropy Analysis

**Figure 10:**
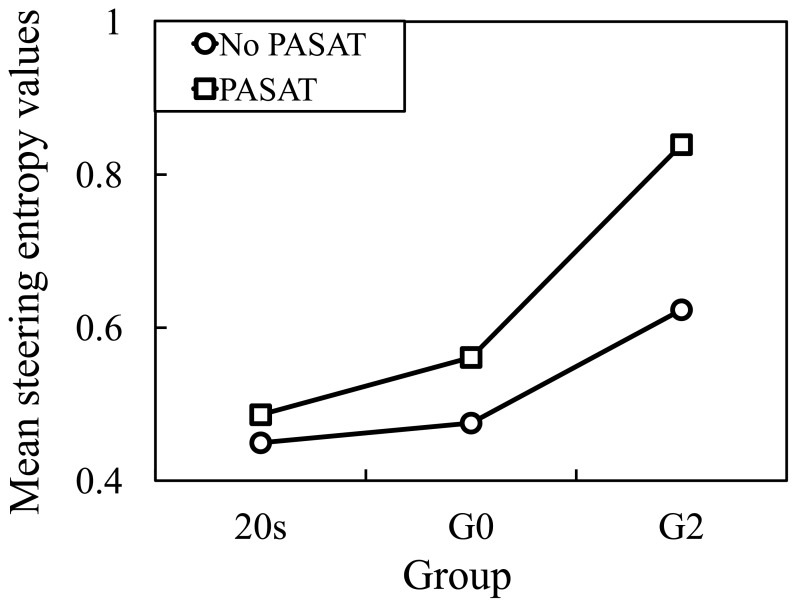
Steering entropy values with and without PASAT for right turns.

The SEVs for right turns are presented in Figure 10. By mixed factorial ANOVA, there were a significant main effect of the Age, *F*(2, 30) = 5.20, *p*<0.05, and a significant main effect of the PASAT, *F*(1, 30) = 181.60, *p*<0.001. Furthermore, there was a significant Age×PASAT interaction, *F*(2, 30) = 27.79, *p*<0.001. By pairwise comparison, values of PASAT were significantly higher than that of No PASAT (*p*<0.001). Additionally, Turkey corrected post hoc tests showed that values of 20 s and G0 did not significantly differ (*p* = 0.80), but values of G2 were significantly higher than both of 20 s and G0 (*p*<0.01 and *p*<0.05).

**Figure 11:**
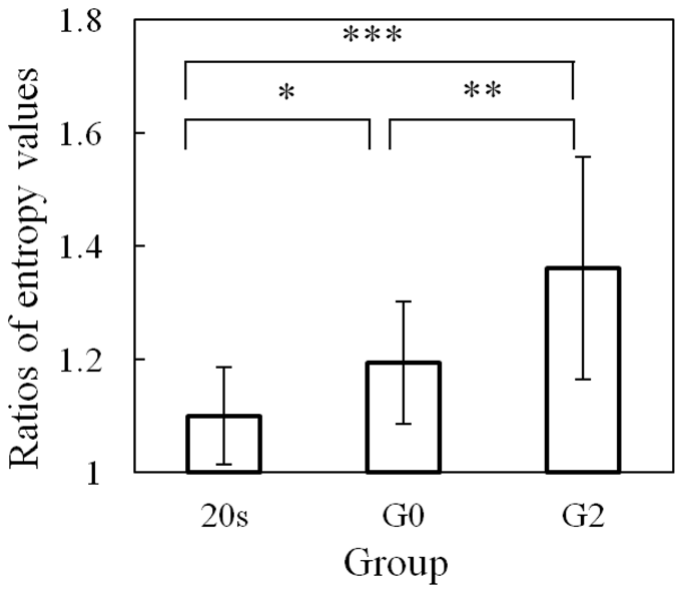
Ratios of steering entropy values for right turns (one-way ANOVA analysis: * *F*(1, 18) = 4.91 and *p*<0.05; ** *F*(1, 22) = 4.50 and *p*<0.05; and *** *F*(1, 20) = 6.21 and *p*<0.05).

The ratios of the SEVs in the conditions with PASAT to those without PASAT are presented in Figure 11. There was a significant difference at the 0.05 level in the ratios of the SEVs between the 20 s and G0, G0 and G2, and 20 s and G2 (one-way ANOVA analysis: F(1, 18) = 4.914 and p<0.05; F(1, 22) = 4.497 and p<0.05; and F(1, 20) = 6.214 and p<0.05). The significant difference between G0 and G2 groups indicates that LA-related effects can result in the impairment of driving performance when making right turns.

**Figure 12:**
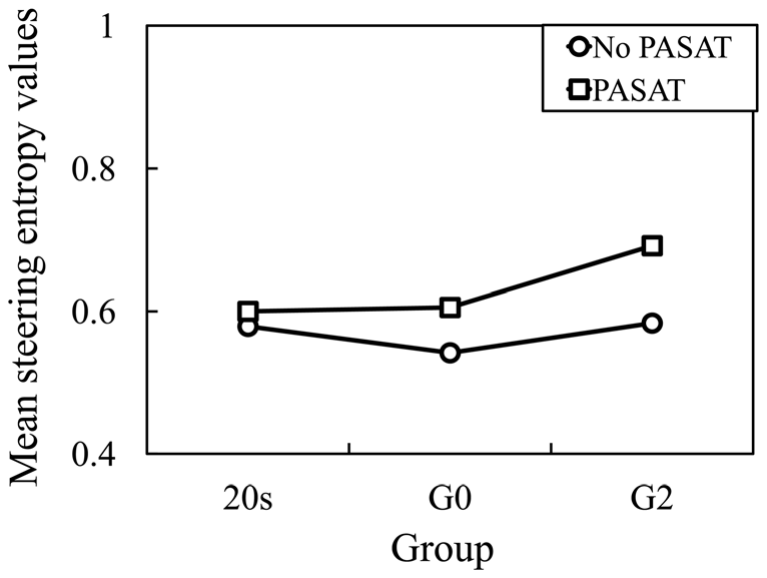
Steering entropy values with and without PASAT for left turns.

The related results of the SEVs for left turns are shown in Figure 12. By mixed factorial ANOVA, there were a significant main effect of the Age, *F*(2, 30) = 7.29, *p*<0.05, and a significant main effect of the PASAT, *F*(1, 30) = 61.50, *p*<0.001. Furthermore, there was a significant Age×PASAT interaction, *F*(2, 30) = 14.57, *p*<0.001. By pairwise comparison, values of PASAT were significantly higher than that of No PASAT (*p*<0.001). Additionally, Turkey corrected post hoc tests showed that values of 20 s and G0, and 20 s and G2 did not significantly differ (*p* = 0.77 and *p* = 0.90), but values of G2 were significantly higher than G0 (*p*<0.05).

**Figure 13:**
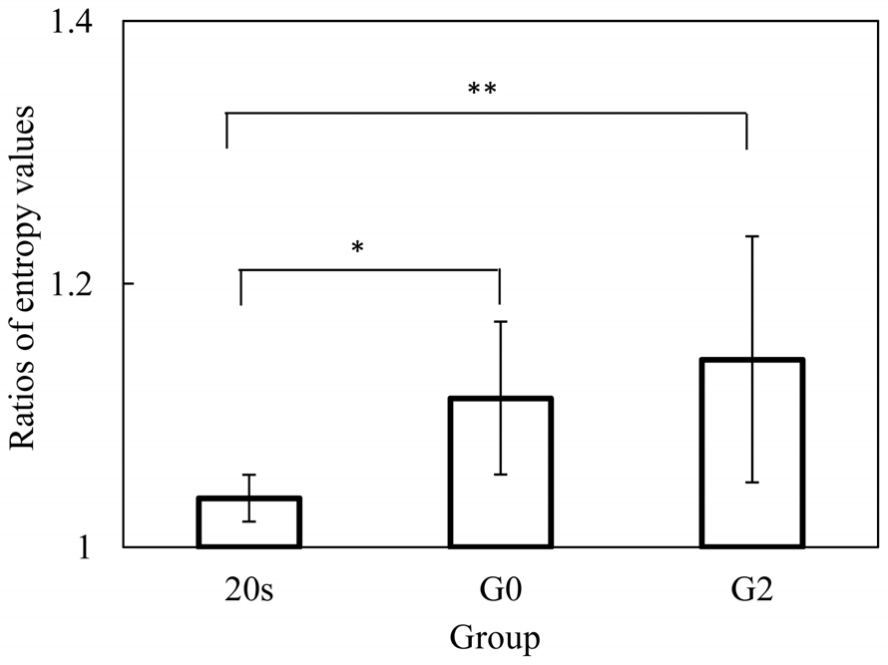
Ratios of steering entropy values for left turns (one-way ANOVA analysis: * *F*(1, 18) = 14.20 and *p*<0.01; and ** *F*(1, 20) = 11.01 and *p*<0.01).

The ratios of the SEVs with PASAT to those without PASAT are shown in Figure 13. There was a significant difference at the 0.05 level in the ratios of the SEVs between 20 s and G0, and between 20 s and G2 (one-way ANOVA analysis: F(1, 18) = 14.202 and p<0.01; and F(1, 20) = 11.010 and p<0.01). The aging-related effect can be observed in the impairment of driving performance when making left turns. However, no significant difference was observed between G0 and G2 groups when making left turnings. Thus, driving task difficulty is an important factor for the LA-related effect for the older drivers, since making left turns is easier than making right turns in Japan, as people drive on the left side of the road.

### Relationship between Rating of Driver Performance and Steering Entropy

**Figure 14:**
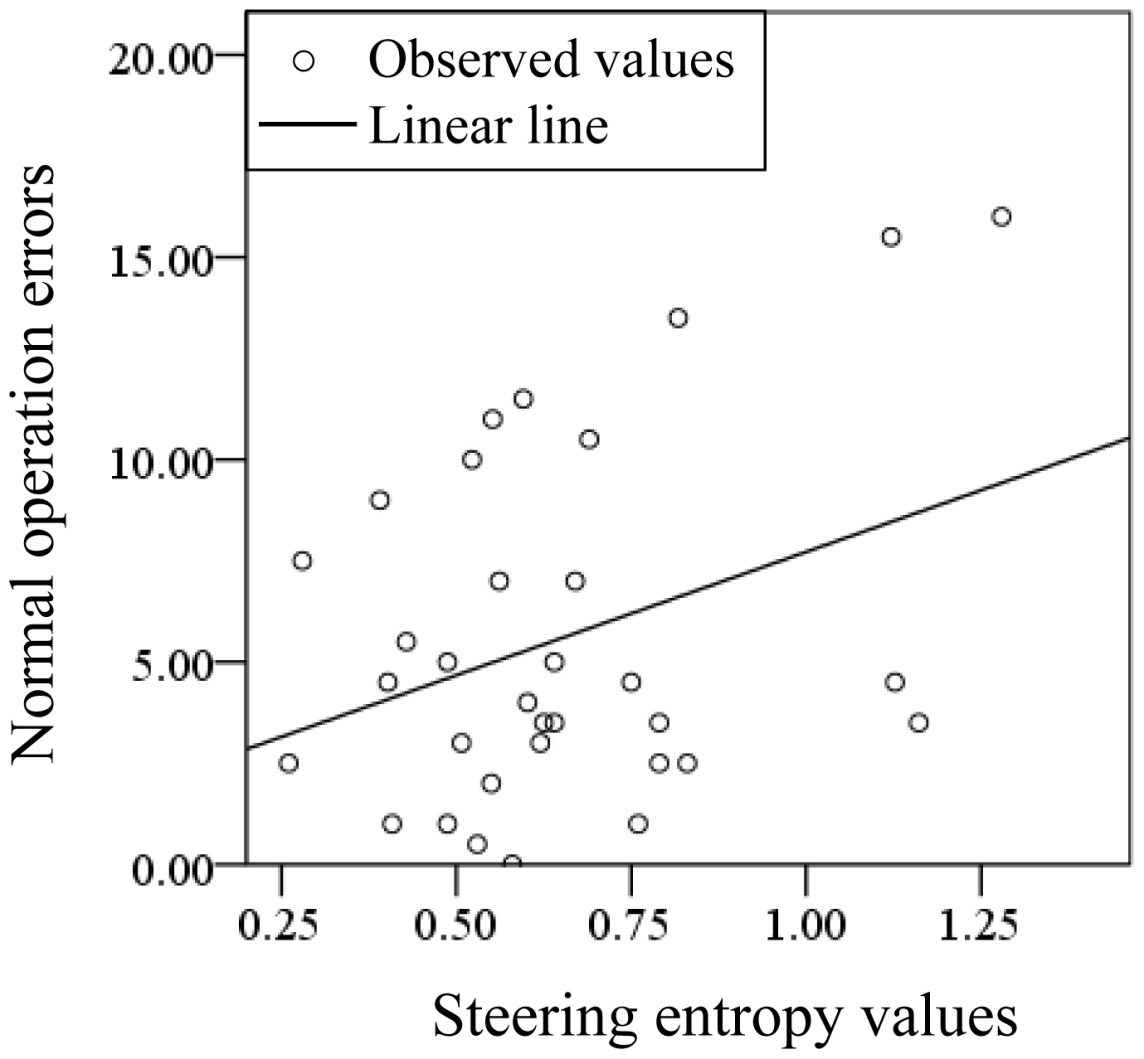
Diagram of regression analysis for making right turns with PASAT.

**Figure 15:**
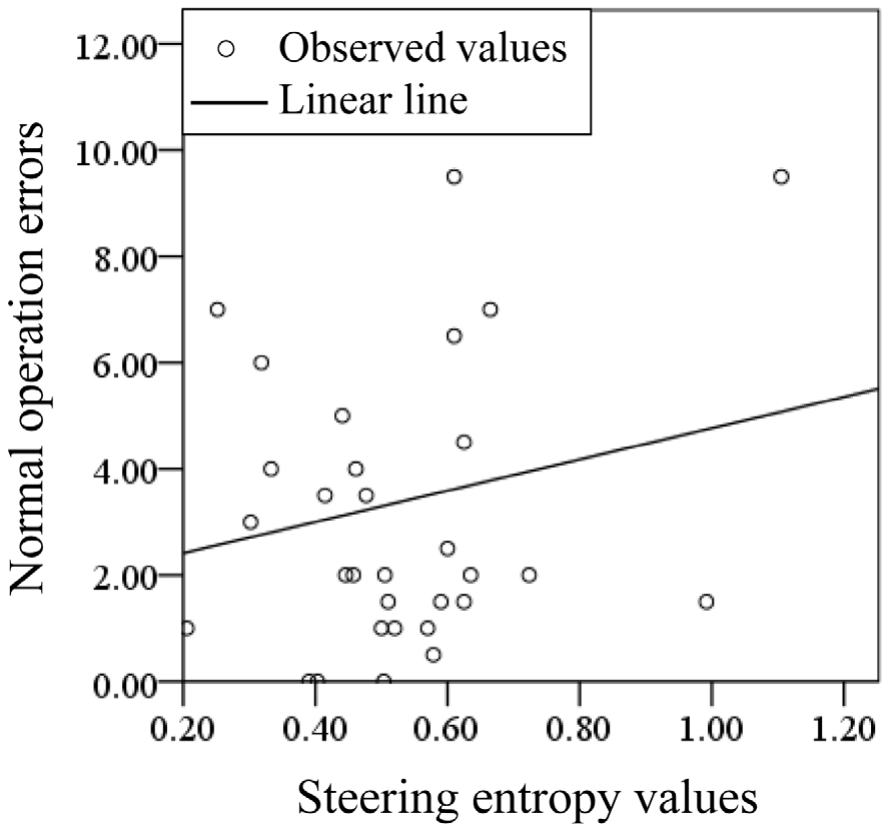
Diagram of regression analysis for making right turns without PASAT.

A regression analysis was carried out to investigate the correlation of SEVs and normal operation error in the study. The regression analysis of SEVs and normal operation error in making right turns with and without PASAT are demonstrated in Figures 14 and 15, respectively. The determination coefficients for the linear lines with and without PASAT were 0.119 and 0.028, respectively. The determination coefficient when PASAT was imposed on the drivers was higher than without PASAT, which suggests that the distraction caused by the PASAT induces rough and unsafe driving.

## Discussion

The motivation of this study is to evaluate the ability of older drivers suffering LA by the comparative analysis of different driver groups. Therefore, both young and older drivers participated in the study, and further the older drivers were divided into two groups depending on their LA progress (G0 and G2 groups). For the correct answer rate of the PASAT, a decrease was found from 20 s to G0 and to G2; however, there was no significant difference between the three groups. Two key points can be extracted from the results. One point is that all participants showed a similar ability to complete the auditory task during the driving experiment, and another is that the ability of the older drivers to respond correctly was so relatively worse than that of the young drivers as to show no significant difference. Importantly, the results rating driver performance demonstrated that a marked difference in driving performance was caused based on the disturbance from the auditory task.

On the analysis of driver performance rating, normal operation errors between the driving conditions with and without the PASAT were significantly different for the 20 s, G0 and G2 groups. However, critical operation errors between the driving conditions with and without the PASAT were significantly different only for the older drivers in the G0 and G2 groups. Further, to analyze the different effects of the PASAT between groups, differences in the operation errors in the conditions with and without the PASAT were calculated for the three groups. For normal operation errors, there was a significant difference between young and older drivers. Furthermore, for critical operation errors, there was a significant difference between the G0 and G2 groups. Critical operation errors such as overlooking stop signs may reflect on more dependence on attention ability than normal operation errors. Because LA exists in the white matter, and is thought to damage the connectivity of neuronal fibers, the attention ability necessary for the prevention of driving operation errors may be reduced.

In terms of the steering operation when turning left and right, the SEVs between the conditions with and without the PASAT were found to be significantly different between the 20 s, G0 and G2 groups for both kinds of behaviors. Corresponding to the driver performance rating, differences between groups based on the effects of the PASAT on steering were also found. A significant difference in the ratios of SEVs exists between G0 and G2 older drivers only for turning right, while the young and older drivers had significant differences in SEVs for turning both left and right. A right-hand-drive automobile has a larger radius of curvature when turning right than left. This larger radius may be reflected in the rougher steering of older drivers with LA. Several papers have reported that the driving performance of older drivers is more easily affected by distraction than that of younger drivers [22]–[24]. As our experiments using PASAT showed a significant difference in driving operation errors and SEVs between the G0 and G2 groups, the existence of LA has a critical effect on driving performance, as does aging. The correlation between the steering entropy and the rating of the driving performance was stronger in driving performance with PASAT than without PASAT. This suggests that distraction during driving induces a lack of smoothness in the driving, resulting in driving operation errors. Bellinger and his colleagues have reported that the movement time from the accelerator to the brake pedal became longer when drivers were distracted [25]. The reason for this may that the motion of drivers becomes quicker to compensate for the delay of the response, resulting in a lack of care and smoothness while operating a vehicle. In other words, the response delay may be linked to distraction, one of several possible declines in cerebral functions due to LA.

It is plausible to consider a significant association between traffic crashes and LA, which as shown by Park et al. [10], is due to vulnerability to distraction for drivers suffering LA. LA may amplify distraction caused by PASAT. As aging is a risk factor in traffic crashes, older drivers suffering LA may be most dangerous drivers apt to cause traffic crashes.

In this study, given the limitations of safety and ethics, the driving experiment was only conducted in the Driver's License Center, without taking surrounding vehicles and real traffic environments into consideration. Therefore, further investigations in real traffic conditions about whether LA causes safe driving performance to deteriorate in older drivers are also necessary for the validation of our results. Meanwhile, we intend to propose and evaluate a reliable driving assistant method in future studies. Additionally, it is an interesting research topic to further investigate G0/G2 differences in older adults while manipulating the difficulty of the secondary task, and a continuous n-back task will be a useful method to compare with the PASAT in this study [26], [27].
